# Prognostic value of dobutamine stress cardiovascular magnetic resonance in patients with previous coronary revascularization

**DOI:** 10.1186/1532-429X-13-S1-P165

**Published:** 2011-02-02

**Authors:** Sebastian Kelle, Rolf Gebker, Amedeo Chiribiri, Juliane Vierecke, Christina Egnell, Ernst Wellnhofer, Christoph Klein, Eckart Fleck

**Affiliations:** 1 German Heart Institute Berlin, Berlin, Germany

## Introduction

The aim of this study was to assess the prognostic value of dobutamine stress cardiovascular magnetic resonance (DCMR) in patients with previous myocardial revascularization. The role of DCMR in the risk stratification of patients with previous coronary revascularization has not been well defined.

## Methods

Clinical data and DCMR results were analyzed in 687 consecutive patients with previous percutaneous or surgical coronary revascularisation undergoing DCMR between 2000 and 2004. Follow up was successful for 654 (95.2%) patients. Two hundred and nineteen patients who underwent early revascularisation (≤3 months) after the test were excluded from analysis. The remaining 435 patients (median age, 64 years) were followed up for a mean of 37 ± 18 months. WMA at rest and the presence of stress-induced WMA (ischemia) were assessed for each patient. Cox proportional hazards regression models were used to identify independent predictors of the composite of cardiac events defined as cardiac death and non-fatal myocardial infarction.

## Results

**Figure 1:**
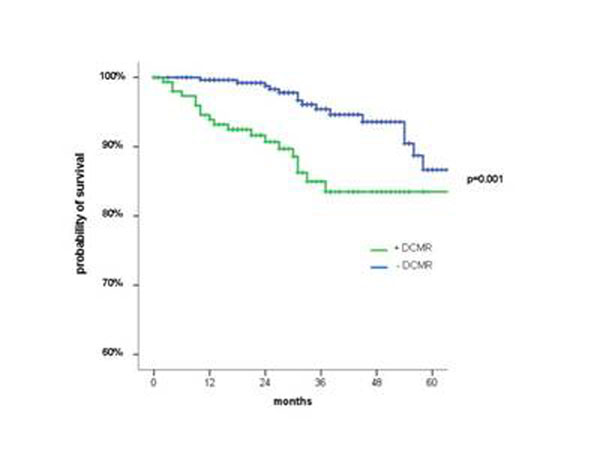
Kaplan-Meier survival curves of patients with previous coronary revascularization with normal and positive DCMR.

Thirty four cardiac events were reported, documented cardiac death in twenty six and non-fatal myocardial infarction in eight patients. In multivariate analysis of clinical data, independent predictors of late cardiac events were the number of dysfunctional segments at rest (hazard ratio (HR) 1.2; 95% confidence interval (CI) 1.1 to 1.3; p<0.001) and stress-induced WMA on DCMR (HR 2.9, 95% CI 1.5 to 5.8; p=0.002). (Figure 1).

## Conclusions

Myocardial ischaemia during DCMR is independently predictive of cardiac events among patients with previous myocardial revascularisation.

